# Supplementary material for: Face mask fit hacks: Improving the fit of KN95 masks and surgical masks with fit alteration techniques
Source: PLoS One. 2022 Feb 2;17(2):e0262830. doi: 10.1371/journal.pone.0262830 (PMC8809580; doi:10.1371/journal.pone.0262830)
Supplement: Attachment — Submitted filename: Comment-7.6.2021.docx [file pone.0262830.s002.docx]

The revised manuscript needs more revision.

1) The lines in the revised MS is not numbered. It’s hard to locate previous comments and the changes made.
2) The reviewer thanks the authors reply. But some of the replies to the reviewer’s comments and edits based on the comments require more work.

**RE: Comments and suggestions to authors’ reply. Replies are labeled as red.**

1) Please note there is a difference between a surgical mask (tie) and a procedural mask

(earloop). I believe the ones tested in this manuscript are procedural masks.

My understanding is that the term surgical mask is an overarching definition which includes

masks which can be secured by tie or earloop. We have now specified that the masks had

earloops and added the term ‘procedural masks’.

Good.

2) Please indicate the models and manufacturers of the masks tested.

This has been added.

Good

3) The abstract could be more concise, especially the “Results and Discussion” section. I also

recommend not using vague words, such as “most” and “many”, repeatedly. How many fit hacks

are effective exactly?

The abstract has been edited to make it more specific.

Good. But numerical presentation of the improved fit score would be better. For example, present percent of improvements in each hack compared with those without would help readers better.

4) Line 65. The claim is not accurate because there are other reasons driving the necessity to

wear a “surgical” mask or a KN95 in the public. For example, one would prefer a “surgical”

mask than N95 because of comfort? In addition, please explain why not testing cloth masks.

They are also widely available to and used by the public.

Each cloth mask has its own shape and there is considerable heterogeneity in the style of these

masks. It would be hard to create generalizable findings. In a separate study, we have assessed

the performance of home-made fabric face masks.

Good.

5) Line 67. Besides fit, material type is also important in filtration efficiency.

This has been noted.

Good

6) The authors have talked a lot about the “fit” of mask and explained what the fit is at line 75. I

would suggest expanding the explanation and mentioning it earlier so that it will help readers to

understand.

This discussion has been expanded.

The reviewer appreciates the authors labeled the changes in red, however, it’s hard to find where the expanded discussion was. it might be even better to help reviewer is to locate where the edits have been made (e.g. page… line…).

7) Line 94-97. I think emphasizing the advantage of quantitative over qualitative fit testing here

is not necessary. If the authors really think these are necessary, I believe they should belong to

the discussion section.

We do think that highlighting the importance of quantitative fit testing in the methodology over

qualitative testing is important as it clarifies to readers that we are not using qualitative methods

that they might have encountered when fit testing masks in hospitals. Healthcare facilities often

use qualitative fit testing, which relies on users saying whether or not they can taste a test

solution which is sprayed around the mask. Since we expect that the article will be of interest to

healthcare workers, we wish to clarify that this method (which they may be familiar with) was

not the method used.

Please specify the qualitative testing (taste of a test solution) in the text then.

8) Line 100. What exactly is the standard formula? I think providing the formula or equation will

really help readers understand how the quantitative method works, including how the fit factor

scores were assigned.

The formula has been added.

Good. But what does each component of the equation means? CB? CA? CR? They should be specified.

9) Line 106: does a Portacount generate real time data output, or just a summary of the whole

test results? Is it only giving a “pass” or “fail” result?

Real time data is available and then a summary is provided with the quantitative fit factor. The

pass/fail result is based on the quantitative result.

Good.

10) Line 110: have the researchers monitored the relative humidity and temperature of the testing

environment? I think reporting these data are important since the relative humidity seems to

affect the particle count significantly.

Temps were between 65* and 75* F and humidity was between 65% and 70%. We have not

included these data in the manuscript as they were not precisely measured at the time of the

study. However, article density is taken into consideration by the machine formula. Fit factor is

calculated by comparing ambient particles and particles in the mask – it is percentage.

I think not measuring and reporting temperature and relative humidity is a limitation of the study. In our experience, the particle count in the low humidity environment is considerably higher than that in the high humidity environment. This will affect the denominator of the equation.

11) Line 115: The test protocol involves breathing, talking, bending over, and turning the head.

Which exact protocol in 29 CFR § 1910.134 was used? Table A-1, A-2, or A-3?

Table A-2

The method in the manuscript is incorrect. The tests in the Table A-2 include bending over, talking, head up-and-down, head left-and-right, not the ones stated in the manuscript.

12) The data based on fit factor score is hard to understand without knowing how it was

calculated and without a good comparing taget. For example, it will help if a N95 with good fit is

tested (assuming >=95% particles were blocked from entry into the mask) and assigned a score.

Please note that this information is present in the manuscript: “When the fit of N95 or FFP3

masks are assessed, a score of at least 100 is required for the mask to be considered to have

adequate fit”

So fit score of 100 is >=95% of filtered particle?

13) Another common hack that could improve the mask performance is using a hook to tie the

two earloops behind the head. Have you considered and tested this?

We are aware of this hack and use it ourselves in the research group… however, we have

unfortunately not yet tested this. Our understanding is that the main purpose hack is used to

improve comfort rather than the fit of the mask.

No. See this ref: Clapp PW, Sickbert-Bennett EE, Samet JM, et al. Evaluation of Cloth Masks and Modified Procedure Masks as Personal Protective Equipment for the Public During the COVID-19 Pandemic. *JAMA Intern Med.* 2021;181(4):463–469. doi:10.1001/jamainternmed.2020.8168.

14) Line 274. I thought this paper was mainly to improve mask performance of the masks

available and worn by the public. Healthcare workers seem to have no N95 shortage now, at least

in the US.

This paper is mainly to help the public, although there is applicability for healthcare workers.

Surgical masks and KN95 masks are commonplace in hospitals around the world, with KN95s

being prominently used by Asian healthcare workers.

Good
