# Supplementary material for: Face mask fit hacks: Improving the fit of KN95 masks and surgical masks with fit alteration techniques
Source: PLoS One. 2022 Feb 2;17(2):e0262830. doi: 10.1371/journal.pone.0262830 (PMC8809580; doi:10.1371/journal.pone.0262830)
Supplement: Attachment — Submitted filename: Response to Reviewers September 2021 .docx [file pone.0262830.s003.docx]

Dear Editor,

Thank you for sending our article for peer review. We appreciate Reviewer 1’s approval of the manuscript for publication and we have addressed Reviewer 2’s outstanding comments as below. Please note that the reviewer’s original comment is in black, our original reply is in green, the reviewer’s response to our reply is in red and our current response is in blue. Please also find enclosed a tracked changes version of the manuscript.

**RE: Comments and suggestions to authors’ reply. Replies are labeled as red.**

3) The abstract could be more concise, especially the “Results and Discussion” section. I also

recommend not using vague words, such as “most” and “many”, repeatedly. How many fit hacks

are effective exactly?

The abstract has been edited to make it more specific.

Good. But numerical presentation of the improved fit score would be better. For example, present percent of improvements in each hack compared with those without would help readers better.

We appreciate the comment, however we do not think that it would be appropriate to present numerical results in the abstract given that there was a high degree of variability and without sufficient context, these results may be misinterpreted.

7) Line 94-97. I think emphasizing the advantage of quantitative over qualitative fit testing here

is not necessary. If the authors really think these are necessary, I believe they should belong to

the discussion section.

We do think that highlighting the importance of quantitative fit testing in the methodology over

qualitative testing is important as it clarifies to readers that we are not using qualitative methods

that they might have encountered when fit testing masks in hospitals. Healthcare facilities often

use qualitative fit testing, which relies on users saying whether or not they can taste a test

solution which is sprayed around the mask. Since we expect that the article will be of interest to

healthcare workers, we wish to clarify that this method (which they may be familiar with) was

not the method used.

Please specify the qualitative testing (taste of a test solution) in the text then.

This has been added to page 4 under “Quantitative fit testing method”

8) Line 100. What exactly is the standard formula? I think providing the formula or equation will

really help readers understand how the quantitative method works, including how the fit factor

scores were assigned.

The formula has been added.

Good. But what does each component of the equation means? CB? CA? CR? They should be specified.

This has been added to the bottom of page 4 under “Quantitative fit testing method”

10) Line 110: have the researchers monitored the relative humidity and temperature of the testing

environment? I think reporting these data are important since the relative humidity seems to

affect the particle count significantly.

Temps were between 65* and 75* F and humidity was between 65% and 70%. We have not

included these data in the manuscript as they were not precisely measured at the time of the

study. However, article density is taken into consideration by the machine formula. Fit factor is

calculated by comparing ambient particles and particles in the mask – it is percentage.

I think not measuring and reporting temperature and relative humidity is a limitation of the study. In our experience, the particle count in the low humidity environment is considerably higher than that in the high humidity environment. This will affect the denominator of the equation.

We have added this to the Methods under the new heading of ‘Setting’

11) Line 115: The test protocol involves breathing, talking, bending over, and turning the head.

Which exact protocol in 29 CFR § 1910.134 was used? Table A-1, A-2, or A-3?

Table A-2

The method in the manuscript is incorrect. The tests in the Table A-2 include bending over, talking, head up-and-down, head left-and-right, not the ones stated in the manuscript.

Apologies, we used the original protocol, before it was modified to be shorter. We have located a reference for the original protocol we followed and explained this in the text with some more detail about the specific activities that were performed. We followed the protocol exactly that has been specified in the text.

12) The data based on fit factor score is hard to understand without knowing how it was

calculated and without a good comparing target. For example, it will help if a N95 with good fit is

tested (assuming >=95% particles were blocked from entry into the mask) and assigned a score.

Please note that this information is present in the manuscript: “When the fit of N95 or FFP3

masks are assessed, a score of at least 100 is required for the mask to be considered to have

adequate fit”

So fit score of 100 is >=95% of filtered particle?

We have clarified this in the manuscript, so that the scores are easier to understand for our readers. As Fit Factor is outside air divided by filtered air, a simplified way of thinking about fit factor is how much cleaner it is than the surrounding air. Fit factor = outside/mask. Thus mask air x fit factor = outside air. If there are 6 particles inside the mask and 600 particles outside the mask, the mask will have a fit factor of 100. If there are 3 or fewer particles inside a mask and 300 particles outside, the mask has a score of 600. In our experience, we found N95 masks which fit to have less than 1 particle inside the mask, despite air concentrations reading over 800 and sometimes up to 2000. This, along with particle size, may factor into OSHA’s 100 minimum standard. We can determine a fit factor of 100 is 100 times cleaner than the outside air. A mask with a fit score of 20 provides the wearer with air 20x cleaner than the outside air. Wen et al give a good explanation of the mathematics in their 2010 paper, now referenced in the article.

13) Another common hack that could improve the mask performance is using a hook to tie the

two earloops behind the head. Have you considered and tested this?

We are aware of this hack and use it ourselves in the research group… however, we have

unfortunately not yet tested this. Our understanding is that the main purpose hack is used to

improve comfort rather than the fit of the mask.

No. See this ref: Clapp PW, Sickbert-Bennett EE, Samet JM, et al. Evaluation of Cloth Masks and Modified Procedure Masks as Personal Protective Equipment for the Public During the COVID-19 Pandemic. *JAMA Intern Med.* 2021;181(4):463–469. doi:10.1001/jamainternmed.2020.8168.

This has been briefly referred to now in the Discussion on Page 12. We did not test this hack.
